# Supplementary material for: Engineered amphiphilic peptides enable delivery of proteins and CRISPR-associated nucleases to airway epithelia
Source: Nat Commun. 2019 Oct 28;10:4906. doi: 10.1038/s41467-019-12922-y (PMC6817825; doi:10.1038/s41467-019-12922-y)
Supplement: Supplementary file 1 — Supplementary Information [file 41467_2019_12922_MOESM1_ESM.pdf]

Supplementary Information: Supplemental Figures and Tables

Engineered Amphiphilic Peptides Enable Delivery of Proteins and CRISPR Associated Nucleases to Airway Epithelia

Sateesh Krishnamurthy[1], Christine Wohlford-Lenane[1], Suhas Kandimalla[1], Gilles Sartre[1], David K. Meyerholz[2], Vanessa Théberge[3], Stéphanie Hallée[3], Anne-Marie Duperré[3], Thomas Del'Guidice[3], Jean-Pascal Lepetit-Stoffaes[3], Xavier Barbeau[3], David Guay[3], Paul B. McCray, Jr.[1]

[1]Department of Pediatrics, Pappajohn Biomedical Institute, University of Iowa, Iowa City, IA 52242

[2]Department of Pathology, University of Iowa, Iowa City, IA 52242

[3]Feldan Therapeutics, Québec, Québec, Canada

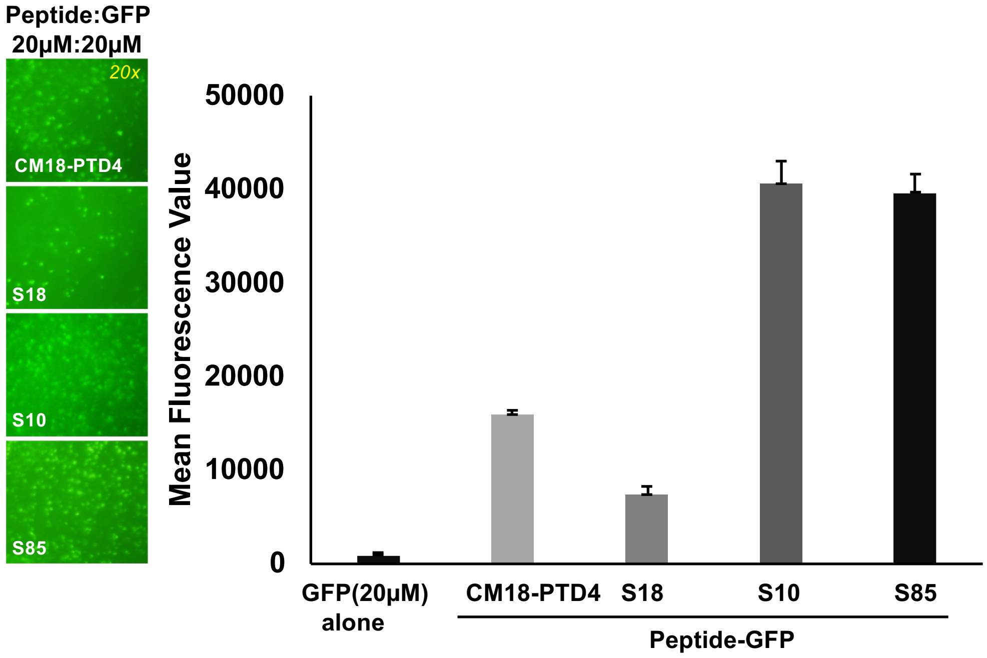

Supplementary Figure 1 Peptide delivery of GFP cargo to well-differentiated primary cultures of porcine airway epithelia. Left panel: representative *en face* views of epithelial sheets following delivery of GFP protein with the indicated four peptides at [peptide]: 20 μM and [GFP]: 20 μM demonstrate GFP^+ cells. 20X magnification. Right panel: Efficiency of all four peptides in delivering GFP protein. Cells were transduced with peptide-GFP combinations (20 μM-20 μM) and GFP signal quantified using a fluorescence reader. Results mean ± SE; n = 4 donors. Data underlying this Figure are provided as Source Data file.

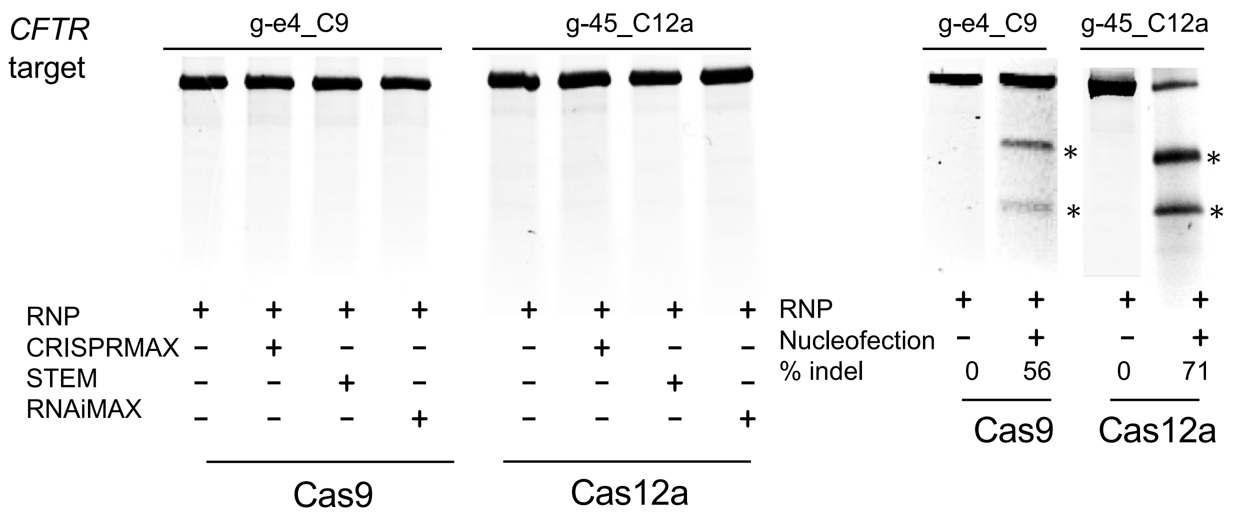

Supplemental Figure 2 Delivery of Cas9 and Cas12a RNPs to well-differentiated primary HAE with three lipofection reagents achieves no editing. **Left panels:** Cas9 and Cas12a RNPs with gRNAs targeting indicated regions in *CFTR* were delivered with 3 different commercially available lipofection reagents as indicated (see Supplementary Table 1 for target regions and guide RNA sequences). 72 hrs later editing was quantified by Surveyor assay. **Right panels:** Positive control Ca9 and Cas12a RNP delivery to well-differentiated primary HAE by electroporation (Amaxa nucleofection) with cells in suspension yields significant editing using the same gRNAs as in Left panels. Data underlying this Figure are provided as Source Data file.

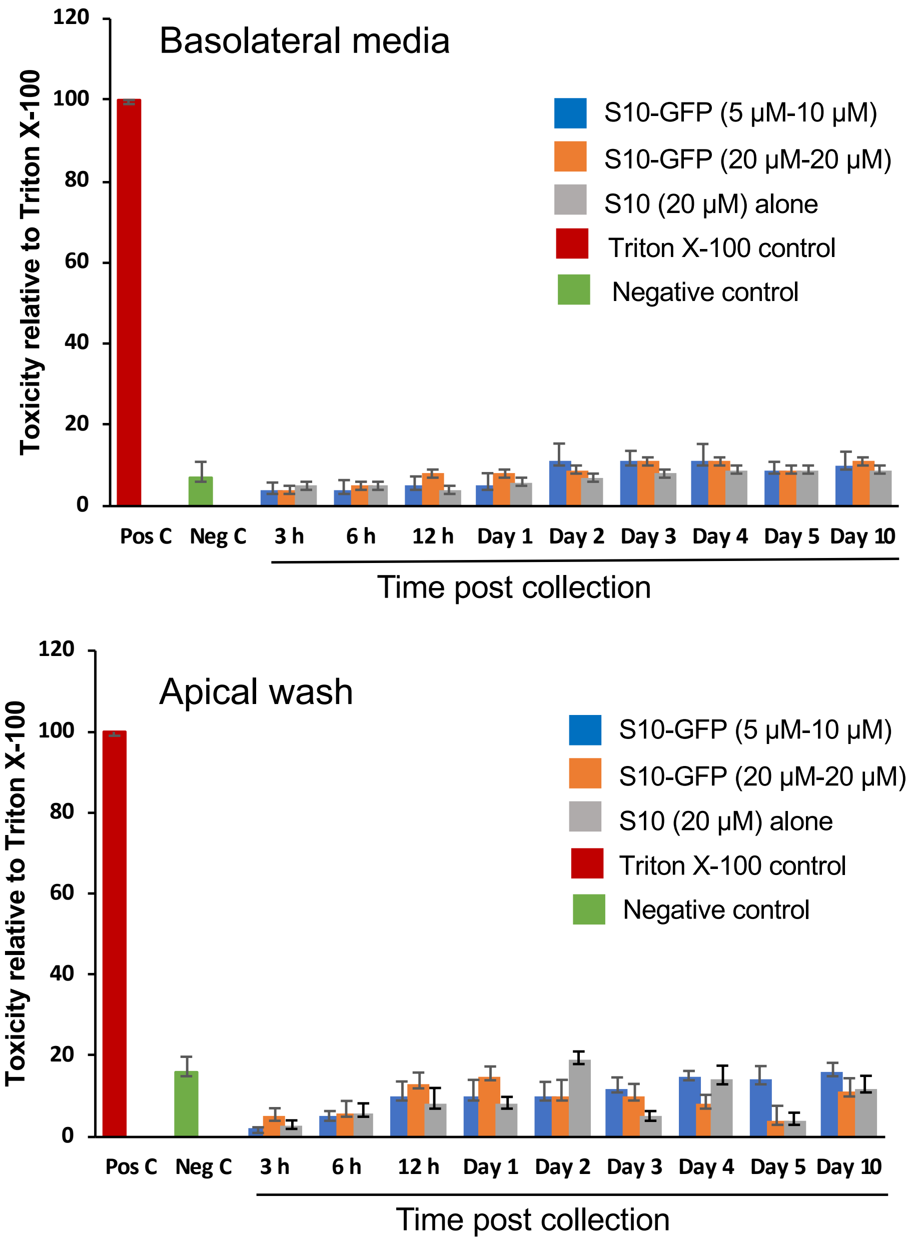

Supplementary Figure 3 *In vitro* toxicity of S10 peptide on human airway epithelia. Well-differentiated cultures of human airway epithelia were treated with S10 peptide and GFP protein at the indicated concentrations. Controls included S10 peptide alone (20 µM), 1% Triton-X 100 detergent in serum free DMEM media (positive control), and serum free DMEM media alone (negative control). Cytotoxicity was assessed by LDH release at the indicated timepoints. Top: LDH release into basolateral media. Bottom: LDH release into airway surface liquid (apical wash). Results mean ± SE; n = 4 donors. Data underlying this Figure are provided as Source Data file.

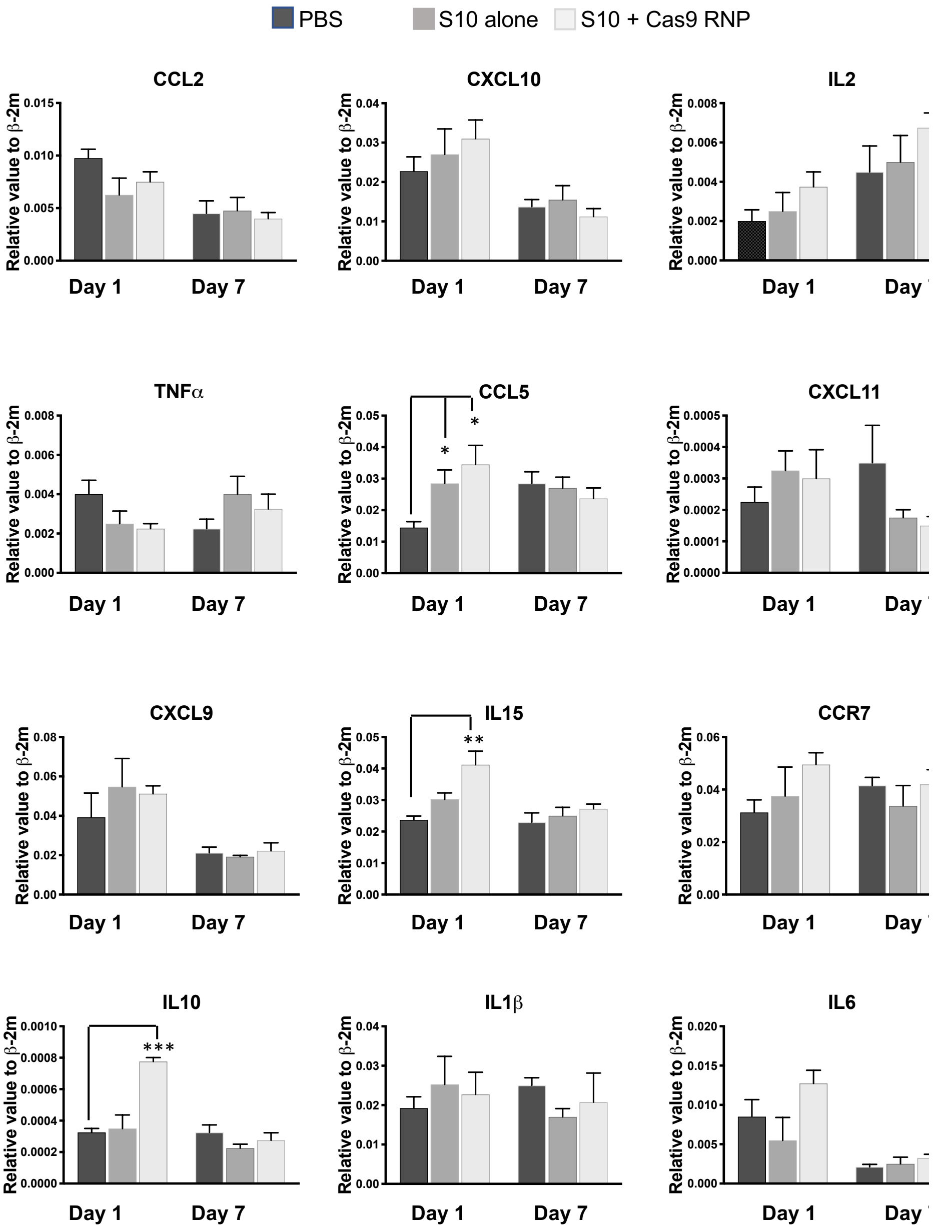

Supplementary Figure 4 Lung tissue mRNA transcript abundance for cytokines and chemokines in mice following S10 peptide and Cas9 RNP administration. RT-qPCR for indicated cytokine and chemokine mRNA expression measured by RT-qPCR on samples collected 1 and 7 days post-delivery (see **Fig. 5**). Results mean ± SE; n = 4mice/group. Data are presented as mRNA abundance normalized to the level of β-2 microglobulin (β-2m). *P<0.05, **P<0.005, ***P<0.001, by one way ANOVA with Tukey's multiple comparison test. Data underlying this Figure are provided as Source Data file.

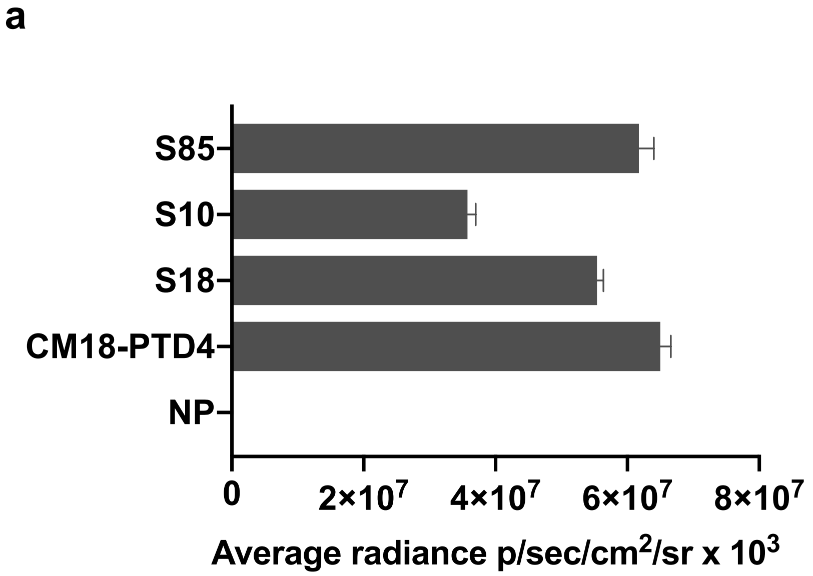

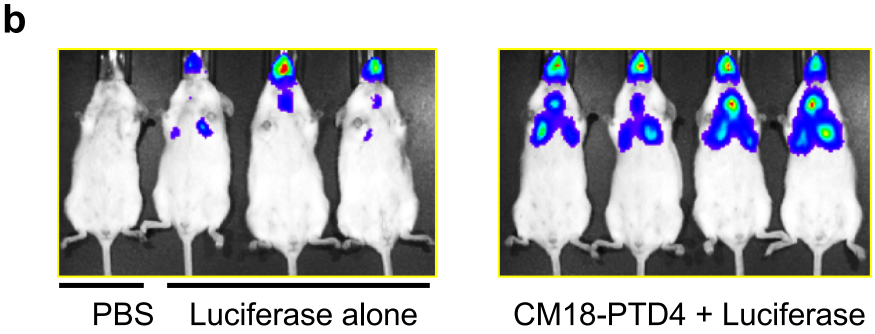

Supplementary Figure 5 (a) Efficacy of 4 peptides in luciferase protein delivery to primary HAE cultures. NP denotes no peptide added. Results mean ± SE; n = epithelia from 4 donors. (b) *In vivo* pulmonary distribution. Mice received CM18-PTD4 peptide (10 μM) and firefly luciferase protein (8 μM) intranasally in a final volume of 50 μl. Control mice received luciferase protein only. Two hours post administration mice received luciferin substrate intraperitoneally, were anaesthetized, and imaged using IVIS system. n = 4 mice/group, representative of 3 replicate experiments. Data underlying this Figure are provided as Source Data file.

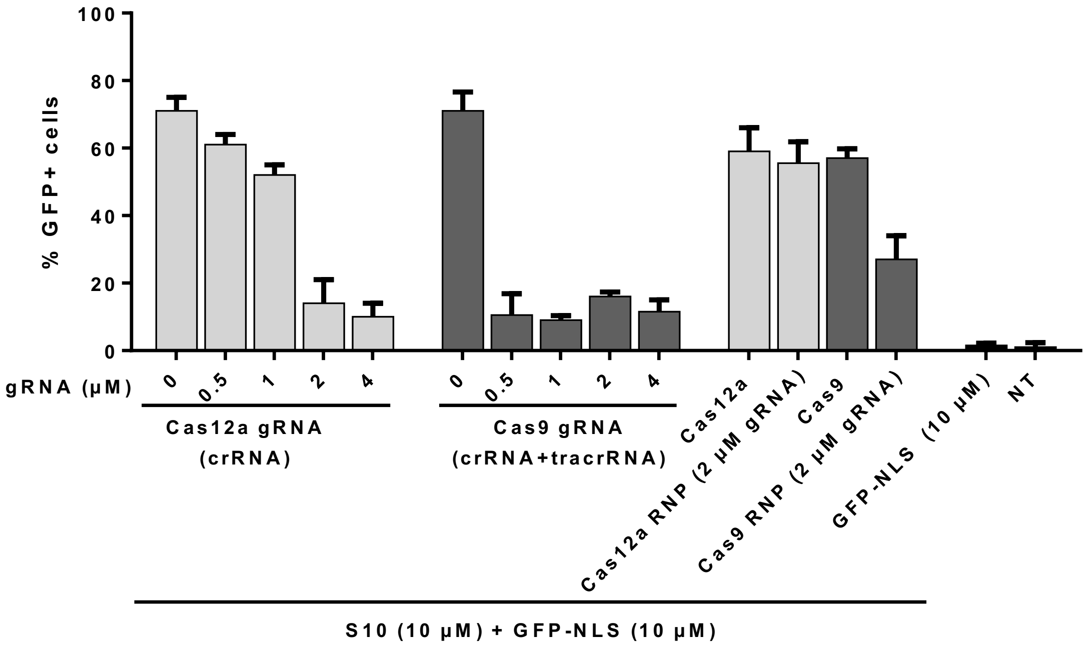

Supplementary Figure 6 Effects of adding Cas12a or Cas9 gRNA alone or Cas12a or Cas9 RNP on S10-mediated GFP protein delivery to HeLa cells. GFP protein (10 μM) and S10 (10 μM) were added to HeLa cells and delivery quantified by FACS (Y-axis). The addition of Cas12a gRNA (crRNA *NKG2A* 0-4 μM) to the transduction mixture resulted in a gradual dose-dependent inhibition of GFP delivery. Addition of Cas9 gRNA (crRNA Ai9+tracrRNA) had a profound inhibitory effect at low concentrations. The right panels show that Cas12 protein alone (1.33 μM) or in RNP form (containing 2 μM gRNA), does not inhibit S10-mediated GFP protein delivery. While Cas9 protein alone (2.5 μM) did not inhibit GFP transduction, Cas9 RNP (containing 2 μM gRNA) inhibited delivery. Far right bars show GFP protein alone and no treatment (NT). Bars indicate mean ± SE, n=2. Data underlying this Figure are provided as Source Data file.

Supplementary Table 1: Cas9 guides

Guide RNA	Target region	Target sequence (20 nt)
g-11_C9	*CFTR* (Exon 11)	5' TCTGTATCTATATTCATCAT
g-e2_C9	*CFTR* (Exon 2)	5' GGTATATGTCTGACAATTCC
g-e4_C9	*CFTR* (Exon 4)	5' TTCCTATGACCCGGATAACA
g-loxP2_C9	ROSA 26 tdT *loxP*	5' CATTATACGAAGTTATATTA
Ai9	Ai9 (stop cassette)	5' AAGUAAAACCUCUACAAAUG

Supplementary Table 2: Cas12a guides

Guide RNA	Target region	Target sequence (21 nt)
NKG2A	*NKG2A* (Exon 3)	5'GGGGCAGAUUCAGGUCUGAGUAG
g-45_C12a	*CFTR* (Intron 22-23)	5'TGGAGACCACAAGGTAATGAA
g-38330_C12a	*HPRT*	5'GGTTAAAGATGGTTAAATGAT
g-loxPbot_C12a	ROSA26 tdT *loxP*	5'GTATAATGTATGCTATACGAA

Supplementary Table 3: Primers used in Surveyor assay

Assay	Forward primer	Reverse primer
Cas12a (NKG2A)	TCACCCTTTTAATTGCACTAGGG	AGCTTCTCTGGAGCTGATGG
Cas12a (g-45_C12a)	CTCTCAAAATGCCTACTGGGAAC	GGCTAGAGTACTTCCCGCA
Cas12a (g-38330_C12a)	ACCATGGTACACTCAGCACG	GAAGTGTCACCCTAGCCTGG
Cas9 (g-11_C9)	GCAAGTGAATCCTGAGCGTG	ACCATTGAGGACGTTTGTCTC
Cas9 (g-e2_C9)	ACAGGTGTAGCCTGTAAGAGAT	TCAGTGTGAAAAATGAGATGTTCC
Cas9 (g-e4_C9)	ATGTAAACTTGTCTCCCACTGTTG	GGGCCTGTGCAAGGAAGTATT

Supplementary Table 4: RT-qPCR primers

Mouse gene	Forward primer	Reverse primer
CCL2	CTTCTGGGCCTGCTGTTCA	CCAGCCTACTCATTGGGATCA
CXCL10	GCCGTCATTTTCTGCCTCAT	GCTTCCCTATGGCCCTCATT
IL2	CCTGAGCAGGATGGAGAATTACA	TCCAGAACATGCCGCAGAG
TNFα	GAACTGGCAGAAGAGGCACT	AGGGTCTGGGCCATAGAACT
CCL5	AGATCTCTGCAGCTGCCCTCA	GGAGCACTTGCTGCTGGTGTAG
CXCL11	GCCCTGGCTGCGATCAT	ACAGCGCCCCTGTTTGAA
CXCL9	GCCATGAAGTCCGCTGTTCT	GGGTTCCTCGAACTCCACACT
L15	CATCCATCTCGTGCTACTTGTGTT	CATCTATCCAGTTGGCCTCTGTTT
CCR7	CCAGCAAGCAGCTCAACATT	GCCGATGAAGGCATACAAGA
IL10	GGTTGCCAAGCCTTATCGGA	ACCTGCTCCACTGCCTTGCT
IL1β	ACTGTTTCTAATGCCTTCCC	ATGGTTTCTTGTGACCCTGA
IL6	GAGGATACCACTCCCAACAGACC	AAGTGCATCATCGTTGTTCATACA
